# Proline-Rich Antimicrobial Peptides in Medicinal Maggots of *Lucilia sericata* Interact With Bacterial DnaK But Do Not Inhibit Protein Synthesis

**DOI:** 10.3389/fphar.2020.00532

**Published:** 2020-04-24

**Authors:** Małgorzata Cytryńska, Mohammad Rahnamaeian, Agnieszka Zdybicka-Barabas, Kristin Dobslaff, Thole Züchner, Guénaël Sacheau, C. Axel Innis, Andreas Vilcinskas

**Affiliations:** ^1^Department of Immunobiology, Institute of Biological Sciences, Maria Curie-Sklodowska University, Lublin, Poland; ^2^Department of Bioresources, Fraunhofer Institute for Molecular Biology and Applied Ecology, Giessen, Germany; ^3^Institute of Bioanalyticappll Chemistry, Faculty of Chemistry and Mineralogy and Center of Biotechnology and Biomedicine, University of Leipzig, Leipzig, Germany; ^4^Department of Bioanalytics and Laboratory automation, Faculty of Life Sciences, Albstadt-Sigmaringen University, Sigmaringen, Germany; ^5^ARNA Laboratory, Inserm U1212, CNRS UMR 5320, Institut Européen de Chimie et Biologie, University of Bordeaux, Pessac, France; ^6^Institute for Insect Biotechnology, Justus-Liebig-University of Giessen, Giessen, Germany

**Keywords:** medicinal maggots, antimicrobial peptides, proline-rich peptides, mode of action, *Lucilia sericata*

## Abstract

In the search for new antibiotics to combat multidrug-resistant microbes, insects offer a rich source of novel anti-infectives, including a remarkably diverse array of antimicrobial peptides (AMPs) with broad activity against a wide range of species. Larvae of the common green bottle fly *Lucilia sericata* are used for maggot debridement therapy, and their effectiveness in part reflects the large panel of AMPs they secrete into the wound. To investigate the activity of these peptides in more detail, we selected two structurally different proline rich peptides (Lser-PRP2 and Lser-PRP3) in addition to the α-helical peptide Lser-stomoxyn. We investigated the mechanism of anti-*Escherichia coli* action of the PRPs *in vitro* and found that neither of them interfered with protein synthesis but both were able to bind the bacterial chaperone DnaK and are therefore likely to inhibit protein folding. However, unlike Lser-stomoxyn that permeabilized the bacterial membrane by 1% at the low concentration (0.25 µM) neither of the PRPs alone was able to permeabilize *E. coli* membrane. In the presence of this Lser-stomoxyn concentration significant increase in anti-*E. coli* activity of Lser-PRP2 was observed, indicating that this peptide needs specific membrane permeabilizing agents to exert its antibacterial activity. We then examined the AMPs-treated bacterial surface and observed detrimental structural changes in the bacterial cell envelope in response to combined AMPs. The functional analysis of insect AMPs will help select optimal combinations for targeted antimicrobial therapy.

## Introduction

The common green bottle fly *Lucilia sericata* is a species of blowfly found in many temperate and tropical regions. The females usually lay eggs in carrion, but also in the skin, necrotic wounds and hair of living animals. The attraction of the larvae to necrotic tissue can lead to myiasis, but for centuries the larvae have also been used as so-called medicinal maggots for the treatment of infected, non-healing wounds (Church, 1996). Maggot debridement therapy was developed as a formal treatment in the 1930s (Ceřovský and Bém, 2014) and currently involves the application of sterile, laboratory-reared larvae to the wound surface, where they remove necrotic tissue, disinfect the wound, and stimulate healing (Sherman et al., 2000; Beasley and Hirst, 2004; Nigam et al., 2006a; Nigam et al., 2006b; Huberman et al., 2007). Maggot therapy helps particularly the patients with diabetes or cardiovascular disease to resolve chronic ulcers and long-lasting infections (Sherman, 2003; Sherman, 2014; Malekian et al., 2019).

The mechanisms of maggot therapy involve a combination of mechanical debridement to remove necrotic tissue and the secretion/excretion of a cocktail of proteases, antimicrobials, and immunomodulatory factors, the latter inhibiting the pro-inflammatory response of human neutrophils that infiltrate the wound area, thus promoting wound healing (van der Plas et al., 2007). However, traditional maggot therapy is often uncomfortable for patients and the maggots have a limited shelf life. Researchers have therefore focused on the identification of active molecules in the larval secretions/excretions and hemolymph, which can suppress the growth of several key human pathogens including *Streptococcus pyogenes*, methicillin-resistant *Staphylococcus aureus* (MRSA), and vancomycin-resistant *Enterococcus* spp. (Beasley and Hirst, 2004; Kerridge et al., 2005; Nigam et al., 2006a; Nigam et al., 2006b).

When applied to wounds, *L. sericata* larvae produce antimicrobial chemicals such as proline dioxopiperazine and *p*-hydroxybenzoic acid (Huberman et al., 2007). In addition, the larvae produce a broad range of antimicrobial peptides (AMPs) and proteins (Bexfield et al., 2004; Altincicek and Vilcinskas, 2009; Čeřovský et al., 2010; Ratcliffe et al., 2011; Pöppel et al., 2014). These have a greater therapeutic potential because their activity profiles can be modified by mutation, and combinations of different AMPs provide the opportunity for beneficial interactions such as additive effects, potentiation, and synergy (Rahnamaeian et al., 2015; Rahnamaeian et al., 2016; Bolouri Moghaddam et al., 2016; Wu et al., 2018). This reduces the concentrations required for effective protection against pathogens when applied in wound dressings (Bulet and Stöcklin, 2005; Vilcinskas, 2013).

Several previous investigations have addressed the AMP repertoire of *L. sericata*. The earliest study used subtractive hybridization to identify 65 genes induced by septic injury, revealing the presence of AMPs representing the defensin and diptericin families as well as three proline-rich peptides (PRPs) with similarities to *Drosophila melanogaster* drosocin and metchnikowin (Altincicek and Vilcinskas, 2009). More recent studies have characterized the defensin-family AMP lucifensin (Andersen et al., 2010; Čeřovský et al., 2010; Čeřovský et al., 2011) and the antifungal AMP lucimycin (Pöppel et al., 2014). Most recently, RNA-Seq transcriptome analysis of the salivary glands, crop and gut of *L. sericata* maggots, which are the tissues most closely involved in synthesis of AMPs secreted into wounds, identified 47 putative AMP genes encoding (i) three members of the attacin family, (ii) eight cecropins and five additional cecropin-like peptides, (iii) a stomoxyn, (iv) two sarcotoxins, (v) eight defensin-like peptides, (vi) five putative homologs of diptericin, (vii) four PRPs, and (viii) 10 so-called edin (elevated during infection) proteins (Pöppel et al., 2015). A selection of 23 AMPs was synthesized and tested against a broad panel of bacteria and fungi, revealing that the cecropins and stomoxyn were particularly active against Gram-negative bacteria, and the PRPs were moderately active against Gram-negative *Escherichia coli* and *Pseudomonas aeruginosa*. Pairwise tests revealed mostly additive effects between stomoxyn and the cecropins/cecropin-like peptides, as well as the synergistic activity of Def4 and Cec6 against *Micrococcus luteus* (Pöppel et al., 2015; Hirsch et al., 2019).

We focused on the *L. sericata* α-helical peptide stomoxyn and two of the PRPs (**Table 1**) because they have distinct antimicrobial spectra and are strongly induced by septic injury (Altincicek and Vilcinskas, 2009). Lser-stomoxyn (41 amino acids) has also been extensively tested along with another AMP (Lser-sarcotoxin) against a panel of 114 multi-drug resistant clinical isolates, and showed activity against clinical isolates of *E. coli*, *Enterobacter* spp., *Klebsiella* spp., *Salmonella enterica*, *Citrobacter freundii*, and *Acinetobacter* spp., as well as multiple isolates of *P. aeruginosa* (Hirsch et al., 2019). The target range of the *L. sericata* PRPs is unclear because the activity of this class of peptides differs according to the peptide length (Otvos, 2002; Wiesner and Vilcinskas, 2010). PRPs contain a conserved domain responsible for general antimicrobial activity and a variable domain that determines the antimicrobial spectrum (Rahnamaeian, 2011). Short-chain PRPs (< 20 residues) are mainly active against Gram-negative bacteria, whereas longer peptides (≥20 residues) are mainly active against Gram-positive bacteria and ascomycete fungi (Levashina et al., 1995; Rahnamaeian et al., 2009; Rahnamaeian and Vilcinskas, 2012). The two *L. sericata* PRPs we tested are classified as long-chain peptides: Lser-PRP2 (38 amino acids) and Lser-PRP3 (34 amino acids). Previously, we found they were both inactive against *M. luteus* and *E. coli* at concentrations of up to 100 µM (Pöppel et al., 2015). PRPs appear to be dependent on pore-forming peptides for their effects, given their weak bactericidal activity when presented alone (Rahnamaeian et al., 2015). The current model is that PRPs enter bacterial cells through pores formed by other AMPs (such as the α-helical stomoxyn) and inactivate the chaperone DnaK by binding to its ATPase domain (Otvos et al., 2000; Kragol et al., 2001; Li et al., 2006). This causes the massive disruption of bacterial protein metabolism by blocking the function of GroEL, leading to defective ribosome biogenesis and the aggregation of large proteins (Wiesner and Vilcinskas, 2010; Calloni et al., 2012).

**Table 1:** *Lucilia sericata* antimicrobial peptides (AMPs) used in this study.

Peptide	Sequence	Peptide type	Reference
Lser-Stomoxyn	GFRKRFNKLVKKVKHTIKETANVSKDVAIVAGSGVAVGAAMG	Canonical α-helical peptide	Pöppel et al., 2015
Lser-PRP2	EWRPHGSIGGSGLRPGRPQTLPPQRPRRPDFNGPRHRF	Proline-rich (proline content ~21%)	Pöppel et al., 2015
Lser-PRP3	SPFVDRPRRPIQHNGPKPRIITNPPFNPNARPAW	Proline-rich (proline content ~26%)	Pöppel et al., 2015

All peptides possess C-terminal amides.

It is currently unclear whether this general model of PRP function is preserved in *L. sericata*. We therefore investigated first the possible mechanism of action of the PRPs by studying their effects *in vitro* on protein synthesis and their interactions with the bacterial chaperone DnaK. Then we tested the antimicrobial properties of Lser-stomoxyn, Lser-PRP2, and Lser-PRP3 alone and in combination and conducted morphological and nanomechanical analysis of the bacterial cell surface to identify detrimental structural alterations in the cell envelope.

## Materials and Methods

### Microorganisms

We tested the activity of the *L. sericata* AMPs against *E. coli* JM83, carrying plasmid pCH110 (Pharmacia-Amersham, Piscatway, NJ, USA).

### Peptide Synthesis and Modification

Lser-stomoxyn, Lser-PRP2, and Lser-PRP3 were synthesized by PANATecs (Tübingen, Germany) at >95% purity with C-terminal amidation (**Table 1**). For the quenching assay, Lser-PRP2 and Lser-PRP3 were synthesized with N-terminal 5(6)-carboxyfluorescein and C-terminal amidation. DnaK was produced by Michael Zahn (Knappe et al., 2011). The Black Hole Quencher 10 succinimidyl ester (BHQ10-NHS ester, > 75% purity) was purchased from BioCat (Heidelberg, Germany).

### Labeling DnaK With BHQ10

DnaK (2 mg/ml) was dialyzed against the modifying buffer (20 mM Na_2_HPO_4_, 20 mM KH_2_PO_4_, 5 mM MgCl_2_, 150 mM KCl; pH 7.4) before labeling with a 10-fold molar excess of BHQ10-NHS ester (Kreisig et al., 2011; Dobslaff et al., 2012). Excess BHQ10 was removed by further dialysis and the final DnaK concentration was adjusted to 4 mM. To evaluate the labeling efficiency, we measured BHQ10 absorption at 515 nm, and calculated the ratio of dye to protein (mean labeling degree, nine BHQ10 molecules per DnaK).

### Fluorescence Resonance Energy Transfer (FRET) Assay

We mixed 50 μl of the fluorescein-modified peptides (1.3 nM) with 50 μl of a 1:4 serial dilution series of DnaK-BHQ10 in modification buffer (0.8–13,000 nM) in a solid black 384-well plate and incubated the plates for 2 h as previously described (Dobslaff et al., 2012). To calculate the quenching effect, control mixtures (50 μl of the peptide solution and 50 μl of the modification buffer) were recorded five times. We recorded the fluorescence intensity on a Paradigm fluorescence reader using a fluorescence (fluo-rhod) detection cartridge (excitation wavelength = 485 ± 10 nm, emission wavelength = 535 ± 12.5 nm, integration time = 140 ms). The quenching effect was expressed as the percentage of the fluorescence intensity of the control quenched after the addition of DnaK-BHQ10.

### Determination of K_d_ Values

Dissociation constants (K_d_) were determined using SlideWrite v7.01. The quenching effects were plotted against the DnaK concentration (logarithmic abscissa). K_d_ values were calculated by non-linear regression using the dose–response logistical transition function of the program [y = a 0 + a 1/(1 + x/a 2) a3].

### Cell-Free Protein Synthesis Inhibition Assay

We examined the effect of the increasing concentrations of *L. sericata* PRPs (0.5–100 µM) on the luminescence produced following the translation of firefly luciferase in an *E. coli* cell free expression system. S30 extract was prepared from *E. coli* KC6 (DE3) Δ*smpB* Δ*ssrA* cells as previously described (Seidelt et al., 2009). The cell-free protein synthesis reaction consisted of S30 extract diluted to a final concentration of 9.9 mg/ml protein, 130 mM potassium glutamate, 10 mM ammonium glutamate, 15 mM magnesium glutamate, 2 mM each of the 20 standard amino acids, 1.2 mM ATP, 0.85 mM each of CTP, GTP, and UTP, 34 μg/ml folinic acid, 1.5 mg/mL total *E. coli* MRE-600 tRNA (Roche Applied Science, Penzburg, Germany), 33 mM pyruvate, 0.33 mM NAD, 0.26 mM coenzyme A, 4 mM sodium oxalate, 1.5 mM spermidine, 1 mM putrescine, 60 mM Bis-Tris acetate (pH 7.0), 100 µg/ml T7 RNA polymerase (Promega, Madison, WI, USA), and 15 µg/ml pIVEX2.3d_luc plasmid to drive the expression of luciferase. The rest of the assay was carried out as previously described for other translation inhibitors (Starosta et al., 2010). Samples were incubated for 2 h at 30°C and the reaction was stopped by adding 2 µM kanamycin and incubating the samples on ice. We then distributed 130 µl luciferase stabilization buffer (70 mM HEPES-KOH pH 7.7, 7 mM MgSO_4_, 3 mM dithiothreitol, 1% bovine serum albumin) into 96-well plates (Greiner Bio-One) and added 20 µl of each sample and 5 µl Steady-Glo Luciferase Assay System (Promega). Luminescence was measured using a Tecan infinite M1000 PRO plate reader. Relative luminescence values were obtained for each sample by setting the luminescence value of the control without inhibitor to 100%.

### Bacterial Membrane Permeabilization Assay

Membrane permeabilization was quantified by measuring the activity of β-galactosidase released from *E. coli* JM83 cells carrying plasmid pCH110, which encodes a constitutive, cytoplasmic form of the enzyme (Zdybicka-Barabas et al., 2013). The peptides were pre-incubated for 15 min at 37°C in 23 μl 20 mM phosphate buffer (pH 6.8) before adding 2 μl of a suspension of mid-logarithmic phase *E. coli* cells (5 × 10^5^ colony forming units, prepared in the same buffer). The final AMP concentrations were 0.0625–2 µM Lser-stomoxyn and up to 50 µM for the Lser-PRPs (Pöppel et al., 2015; Hirsch et al., 2019). After incubation for 45 min at 37°C, we added 220 μl 20 mM HEPES/150 mM NaCl (pH 7.5) and 5 μl 50 mM *p*-nitrophenyl-β-D-galactopyranoside. We incubated the samples at 37°C for a further 90 min and then measured the absorbance at 405 nm, which is proportional to the amount of released β-galactosidase. Live bacteria incubated in growth medium as well as dead bacteria after treatment with 5 µM synthetic cecropin B (Sigma-Aldrich) served as control samples. We set the perforation level of dead bacteria to 100%. All assays were performed six times in triplicate for each type of sample.

### Atomic Force Microscopy

Bacterial samples were prepared for atomic force microscopy (AFM) as previously described (Zdybicka-Barabas et al., 2012; Zdybicka-Barabas et al., 2013). Briefly, log-phase *E. coli* JM83 cells (OD_600_ = 0.2) in 100 µL lysogeny broth were incubated at 37°C for 90 min in the presence of individual or combined AMPs, or without AMPs as a negative control. We used concentrations of 50 μM Lser-PRP2, 50 μM Lser-PRP3, and 0.25 μM Lser-stomoxyn. The samples were centrifuged (8,000 × g, 4°C, 10 min), washed twice with apyrogenic water, resuspended in 5 μl apyrogenic water, applied to mica disks and allowed to dry at 28°C overnight.

The cell surface was imaged using a NanoScope V AFM (Veeco, Plainview, NY, USA) in Peak Force QNM operation mode and a silicon tip NSG 30 with a spring constant of 20 N/m (NT-MDT, Moscow, Russian Federation). The results were processed using Nanoscope Analysis v1.40 (Veeco). Three fields on each mica disk were imaged. The roughness values were measured over the entire bacterial cell surface in 3 × 3 µm areas. The average surface root mean square (RMS) roughness was calculated from 25 fields (300 × 300 nm). Section profiles and 3D images were produced using WSxM v5.0 (Horcas et al., 2007).

## Results

### Mechanisms of Action of Lser-PRPs—*In Vitro* Study

PRPs are generally unable to pass through an intact cell membrane, and require either a receptor molecule or the co-presentation of pore-forming AMPs in order to reach the cytoplasm. Once they have crossed the membrane, they can interact with one of two specific targets. Either they interact with ribosomes to directly inhibit protein synthesis, or they interact with DnaK to interfere with protein folding (Castle et al., 1999; Otvos et al., 2000; Kragol et al., 2001; Rahnamaeian, 2011; Czihal et al., 2012).

**Figure 1:**
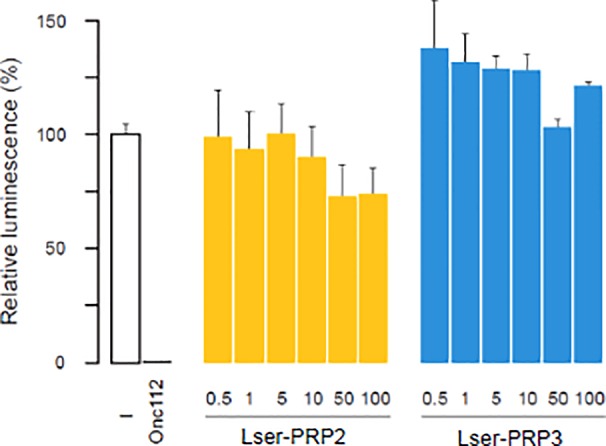
Effect of *Lucilia sericata* proline rich peptides (PRPs) on bacterial protein synthesis. The graph shows the effects of increasing concentrations of Lser-PRP2 (yellow) and Lser-PRP3 (blue) on the luminescence produced following the translation of firefly luciferase in an *Escherichia coli* cell free expression system. Data are means ± standard deviations (**n** = 3) and the luminescence was normalized relative to that measured in the absence of peptide, which was set to 100%. The negative sample (white) indicates a control translation reaction performed in the absence of additional peptide. The concentration of peptide used in the Onc112 control was 10 µM.

To determine the possible intracellular targets of Lser-PRP2 and Lser-PRP3, we carried out a cell-free protein synthesis inhibition assay in the presence of increasing concentrations of the peptides (**Figure 1**). The positive control PRP Onc112 inhibited translation effectively, with 10 µM of the peptide abolishing the process entirely, based on inhibition of production of luciferase. We found that Lser-PRP2 at concentrations in the range 0.5–10 µM had no significant impact on luminescence, but increasing the concentration to 50 µM or more caused a ~30% decrease in luminescence relative to the untreated control. These concentrations are nearly two orders of magnitude greater than the IC_50_ value of Onc112 and other PRPs (Seefeldt et al., 2015) so it is unlikely that Lser-PRP2 primarily functions as a translational inhibitor. Similarly, we did not observe any significant variation in luminescence in response to the presence of Lser-PRP3 in the concentration range 0.5–100 µM, and its low concentrations even resulted in a reproducible ~25% increase in luminescence.

**Figure 2:**
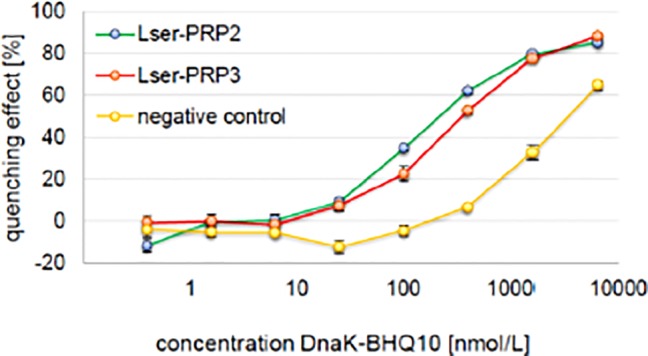
Quenching efficiency of *Lucilia sericata* proline rich peptides (PRPs) in comparison to the negative control apidaecin 1b (9–18) in the presence of increasing concentrations of BHQ10. The DnaK-binding curve of Lser-PRP2 and Lser-PRP3 appeared sigmoidal. As a result, the effective quenching (with maximum quenching effect of >80%) of fluorescence is achieved by the BHQ10 fluorophore indicating the effective interaction of both peptides with bacterial DnaK, which can lead to interference with DnaK functions. Data are means ± standard deviations (**n** = 3).

Accordingly, we next determined the DnaK-binding affinities of Lser-PRP2 and Lser-PRP3 using a FRET-based assay, with apidaecin 1b (9–18) (Cf-PQPRPPHPRL-OH) as a negative control (Dobslaff et al., 2012). The background quenching effect of the negative control with increasing concentrations of DnaK-BHQ10 is visible in **Figure 2**. DnaK-interacting peptides should achieve significantly higher quenching efficiency than the control. The DnaK-binding curves for both Lser-PRP2 and Lser-PRP3 appeared sigmoidal, with maximum quenching effects of 85–88% (**Figure 2**).

The K_d_ values were determined by non-linear regression (Dobslaff et al., 2012) with the best values of 0.14 ± 0.01 µM for Lser-PRP2 and 0.3 ± 0.006 µM for Lser-PRP3 (**Table 2**). This assay is ideal for the identification of peptides with medium and strong binding affinities. The K_d_ values of Lser-PRP2 and Lser-PRP3 were comparable to those of other DnaK-interacting PRPs including native oncocin, pyrrhocoricin derivatives, and bumblebee abaecin, all of which have K_d_ values of ~0.1 µM (Dobslaff et al., 2012; Rahnamaeian et al., 2015). These results clearly show that both Lser-PRP2 and Lser-PRP3 interact with DnaK and this is likely to be the basis of the mechanism of action.

**Table 2:** Sequences of *Lucilia sericata* proline rich peptides with N-terminal modification with 5(6)-carboxyfluorescein and C-terminal amidation (NH_2_).

	Sequence	K_d_ value
Lser-PRP2	EWRPHGSIGGSGLRPGRPQTLPPQRPRRPDFNGPRHRF	0.14 ± 0.01 µmol/L
Lser-PRP3	SPFVDRPRRPIQHNGPKPRIITNPPFNPNARPAW	0.3 ± 0.006 µmol/L

The calculated Kd values are based on the quenching assay.

### Microbial Membrane Permeabilization Activity of *L. sericata* AMPs

**Figure 3:**
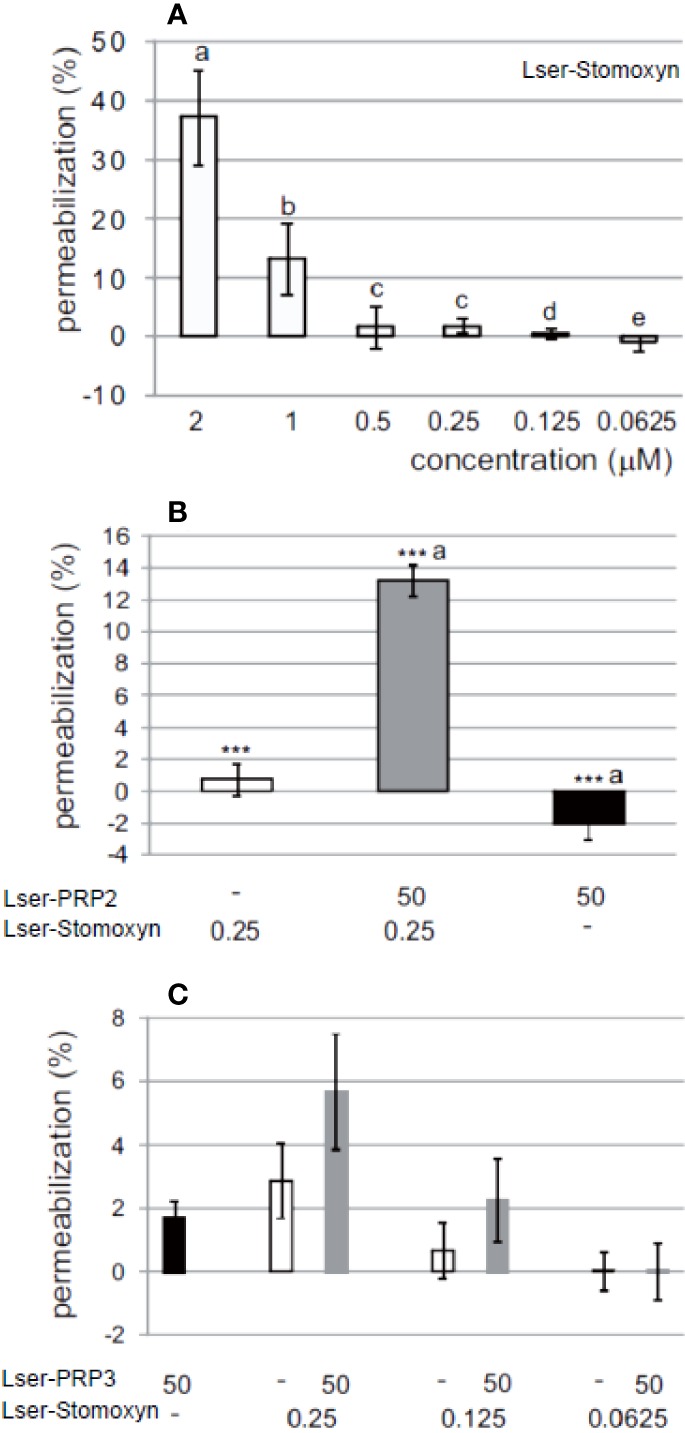
Membrane permeabilization assay. *Escherichia coli* were incubated alone or in the presence of Lser-stomoxyn **(A)** and/or Lser-PRP2 **(B)** and/or Lser-PRP3 **(C)** for 45 min at 37°C. Membrane permeabilization was evaluated by measuring β-galactosidase activity. Live bacteria incubated alone and bacteria killed by exposure to 5 µM synthetic cecropin B served as the control samples. The permeabilization level of the dead bacteria was set to 100%. Data are means ± standard deviations (**n** = 6). **(A)** Statistically significant differences are indicated using different letters (p ≤ 0.001; one-way ANOVA). **(B)** The same letters indicate statistically significant differences between the peptide-treated experimental groups (Mann-Whitney U test). The asterisks indicate statistically significant differences between peptide-treated bacteria and controls (***p ≤ 0.001).

As expected, neither of the PRPs was able to induce bacterial membrane permeabilization even at concentration of 50 µM (**Figures 3B, C**), in contrast to Lser-stomoxyn used at the concentration range 0.0625–2 µM. There was little evidence of permeabilization by Lser-stomoxyn at concentrations of 0.0625 or 0.125 µM, but 1–2% permeabilization was observed at concentrations of 0.25–0.5 µM, and this increased to 12% at 1 µM and 40% at 2 µM (**Figure 3A**). Of note, in the presence of low concentration of Lser-stomoxyn (0.25 µM), a significant increase in anti-*E. coli* activity of Lser-PRP2 was observed, reflected by increase of membrane permeabilization to ~13% (**Figure 3B**), clearly demonstrating that this peptide requires a compromised membrane to effectively act against bacteria. Interestingly, the mixture containing Lser-PRP3 did not achieve a significant increase in membrane permeabilization, indicating that the synergistic activity between Lser-stomoxyn and PRPs in terms of membrane permeabilization is not universal, but is restricted to specific pairwise interactions.

### AFM Imaging of the *E. coli* Cell Surface Following Exposure to *L. sericata* AMPs

**Figure 4:**
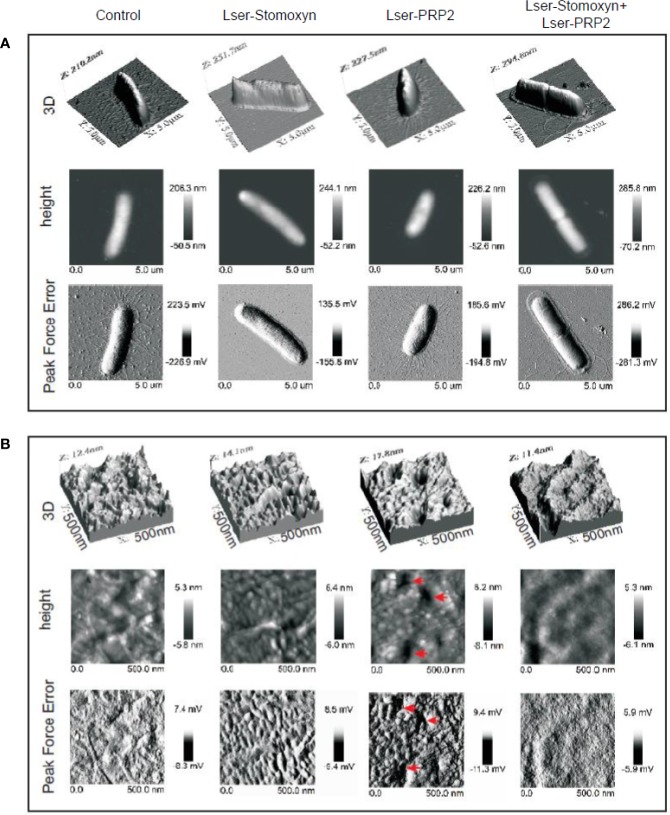
Surface modifications of *Escherichia coli* JM83 cells. The bacteria were incubated alone (control) or in the presence of Lser-stomoxyn (0.25 µM), Lser-PRP2 (50 µM), or a combination of both peptides, before imaging by atomic force microscopy. Three dimensional (3D), height and peak force error 5×5 µm **(A)** and 500×500 nm **(B)** images of the bacteria are presented. In **(B)**, the red arrows in the height and peak force error images indicate recesses observed after treatment with Lser-PRP2.

**Figure 5:**
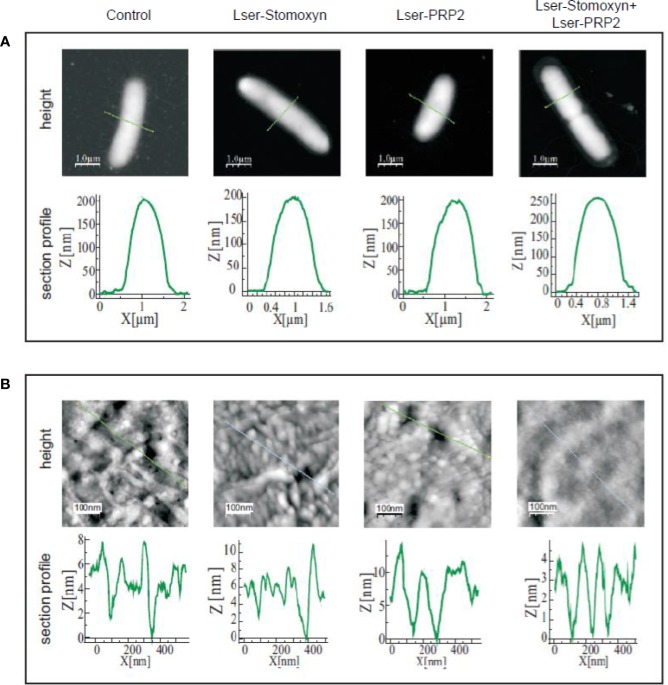
Section profiles of the *Escherichia coli* JM83 cell surface after treatment with Lser-stomoxyn and Lser-PRP2. The bacteria were incubated alone (control) or in the presence of Lser-stomoxyn (0.25 µM), Lser-PRP2 (50 µM), or a combination of both peptides, before imaging by atomic force microscopy. The height 5×5 µm **(A)** and 500×500 nm **(B)** images of the bacterial cell surface are shown as in **Figure 4**. The lower panels indicate the section profiles corresponding to the lines marked in the upper panels. The bars represent 1 µm (upper panels) and 100 nm (lower panels).

To investigate the interactions between Lser-stomoxyn and Lser-PRP2 in more detail, we examined the surface of bacterial cells exposed to the peptides individually and in combination. Untreated control bacteria were rod-shaped with clearly visible flagella, and the cell surface was decorated with small granules and irregular long flat grooves (**Figure 4**). This typical morphology was also observed in our previous studies (Zdybicka-Barabas et al., 2012; Rahnamaeian et al., 2015; Rahnamaeian et al., 2016). The surface of the cells treated with Lser-stomoxyn (0.25 µM) or Lser-PRP2 (50 µM) alone was considerably more granular than that of the controls, and Lser-PRP2 in particular induced the appearance of irregular granules and more numerous recesses with a depth of ~10 nm (**Figure 5**). Interestingly, the cells exposed to Lser-stomoxyn also lost their flagella. In contrast, the surface of cells treated with the combination of both peptides was much less granular, and the recesses were 4–5 nm deep (**Figure 5B**). In addition, the flagella were considerably longer than those of the control cells (**Figure 4**). The morphological changes induced by the AMPs were accompanied by changes in cell surface properties, including an increase in roughness following the treatments with the individual peptides, and an increase in adhesion forces in the cells exposed to the individual peptides and the combination of both peptides (**Table 3**).

**Table 3:** The effect of *Lucilia sericata* antimicrobial peptides used alone and in combination on the *E. coli* JM83 cell surface.

	Control	Lser-Stomoxyn (0.25 µM)	Lser-PRP2 (50 µM)	Stomoxyn (0.25 µM) + Lser-PRP2 (50 µM)
Roughness [nm]	0.843 (± 0.22)	1.102 (± 0.373)^b^***	1.002 (± 0.354)^a^*	0.856 (± 0.295)^ab^
Young's modulus [MPa]	2458 (± 24.52)	2601 (± 32.19)**	2166.14 (± 480.23)	2791.5 (± 429.81)
Adhesion forces [nN]	0.174 (± 0.04)	0.236 (± 0.079)^a^***	0.306 (± 0.160)***	0.3541 (± 0.169)^a^***

The bacteria were incubated alone (control) or in the presence of Lser-stomoxyn (0.25 µM) or Lser-PRP2 (50 µM), or both and then analyzed by atomic force microscopy. The results are presented as means of 25 fields from each sample ± standard deviation (SD). The same letters indicate statistically significant differences between the peptide-treated experimental groups (Mann-Whitney U test). The asterisks indicate statistically significant differences of the peptide-treated bacteria compared to controls (*p < 0.05; **p ≤ 0.01; ***p ≤ 0.001).

## Discussion

The antimicrobial mode of action of insect PRPs is not fully understood. The current model proposes that they target intracellular processes after traversing the cell membrane, which can be facilitated by the presence of pore-forming AMPs (Otvos et al., 2000; Kragol et al., 2001; Li et al., 2006). To shed more light in this process, we investigated the mode of action of two PRPs from *L. sericata* (Lser-PRP2 and Lser-PRP3) in combination with the pore-forming α-helical AMP Lser-stomoxyn.

In order to determine the intracellular targets of Lser-PRP2 and Lser-PRP3, we carried out a cell-free protein synthesis assay and measured the DnaK-binding affinities of both peptides. Our results clearly showed that neither Lser-PRP2 nor Lser-PRP3 have a significant impact on protein synthesis and are therefore unlikely to target the bacterial ribosomal machinery. However, both PRPs bound DnaK with the K_d_ values comparable to those of other DnaK-interacting PRPs including native oncocin, pyrrhocoricin derivatives and bumblebee abaecin, all of which have K_d_ values of ~0.1 µM (Dobslaff et al., 2012; Rahnamaeian et al., 2015). These results clearly demonstrate that both Lser-PRP2 and Lser-PRP3 interact with DnaK, and accordingly this is likely that negative impact on proper protein folding can be the basis of antibacterial mechanism of action of these *L. sericata* peptides.

We previously showed that both PRPs were inactive against the Gram-negative bacterium *E. coli* when used alone (Pöppel et al., 2015) and according to that no bacterial membrane permeabilizing activity was observed in the present study. However, the anti-*E. coli* effect of Lser-PRP2 was significantly enhanced by Lser-stomoxyn, but this was not the case for Lser-PRP3, indicating that the synergistic permeabilization activity of Lser-stomoxyn and PRPs is restricted to specific pairwise interactions. Synergistic interactions have previously been reported between the pore-forming peptide hymenoptaecin and the PRP abaecin in bumblebees, and between gallerimycin and cecropins in the greater wax moth (Rahnamaeian et al., 2015; Bolouri Moghaddam et al., 2016). Functional interactions between different AMPs may therefore be a global strategy to boost the efficacy of AMP arsenals at low concentrations (Rahnamaeian et al., 2016).

## Conclusions

Insect-derived AMPs are promising therapeutic candidates because they possess a wide range of antimicrobial activities, even targeting antibiotic-resistant bacteria such as MRSA (Yi et al., 2014; Rahnamaeian and Vilcinskas, 2015; Mylonakis et al., 2016; Tonk et al., 2016; Tonk and Vilcinskas, 2017). Their potency is enhanced by potentiating and synergistic interactions among peptides with different structural and functional properties. It is therefore important to characterize the functions of AMPs in order to select appropriate complementary activities. Here, we demonstrated that although neither Lser-PRP2 nor Lser-PRP3 were active against *E. coli*, combination of Lser-PRP2 with low concentrations of the pore-forming AMP Lser-stomoxyn lead to an anti-*E. coli* activity reflected by increased permeabilization ability. This combination caused detrimental structural changes in the bacterial cell envelope but the damage caused by the PRP was not enough for antibacterial activity unless Lser-stomoxyn was also present. We also found that both Lser-PRP2 and Lser-PRP3 are likely to function by interacting with DnaK, suggesting that they act by interfering with protein folding rather than directly inhibiting protein synthesis.

## Data Availability Statement

All datasets generated for this study are included in the article/supplementary material.

## Author Contributions

MR designed the experiments and supervised the study. MC, MR, AZ-B, GS, and KD carried out the experiments and analyzed the data. TZ, CI, and AV contributed to materials and reagents. MC, MR, GS, and AV wrote the manuscript.

## Funding

We acknowledge financial support provided by the Hessian Ministry of Science and Art, including a generous grant for the LOEWE research focus “Insect Biotechnology” to AV, as well as the Federal Ministry of Education and Research (BMBF) for Go-Bio funding #0315988 to TZ. The AFM analysis was carried out using equipment purchased with financial support from the European Regional Development Fund in the framework of the Polish Innovation Economy Operational Program (contract no. POIG.02.01.00-06-024/09, Center of Functional Nanomaterials). CI and GS have received funding for this project from the European Research Council under the European Union’s Horizon 2020 research and innovation programme (grant no. 724040). CI is a European Molecular Biology Organization Young Investigator.

## Conflict of Interest

The authors declare that the research was conducted in the absence of any commercial or financial relationships that could be construed as a potential conflict of interest.
